# Intrinsically Disordered Osteopontin Fragment Orders During Interfacial Calcium Oxalate Mineralization

**DOI:** 10.1002/anie.202105768

**Published:** 2021-07-16

**Authors:** Hao Lu, David Yuen Wah Ng, Ingo Lieberwirth, Tobias Weidner, Mischa Bonn

**Affiliations:** ^1^ Department of Molecular Spectroscopy Max Planck Institute for Polymer Research Ackermannweg 10 55128 Mainz Germany; ^2^ Department of Chemistry Aarhus University Langelandsgade 140 8000 Aarhus C Denmark

**Keywords:** calcium oxalate mineralization, interface, kidney stone, osteopontin, sum-frequency generation spectroscopy

## Abstract

Calcium oxalate (CaC_2_O_4_) is the major component of kidney stone. The acidic osteopontin (OPN) protein in human urine can effectively inhibit the growth of CaC_2_O_4_ crystals, thereby acting as a potent stone preventer. Previous studies in bulk solution all attest to the importance of binding and recognition of OPN at the CaC_2_O_4_ mineral surface, yet molecular level insights into the active interface during CaC_2_O_4_ mineralization are still lacking. Here, we probe the structure of the central OPN fragment and its interaction with Ca^2+^ and CaC_2_O_4_ at the water–air interface using surface‐specific non‐linear vibrational spectroscopy. While OPN peptides remain largely disordered in solution, our results reveal that the bidentate binding of Ca^2+^ ions refold the interfacial peptides into well‐ordered and assembled β‐turn motifs. One critical intermediate directs mineralization by releasing structural freedom of backbone and binding side chains. These insights into the mineral interface are crucial for understanding the pathological development of kidney stones and possibly relevant for calcium oxalate biomineralization in general.

## Graphical abstract

Surface‐specific vibrational spectroscopy was applied to study the structure and interaction of central urinary osteopontin peptide at the air–water interface and during calcium oxalate mineralization. The interfacial peptides show ordered and assembled β‐turn motifs with binding of Ca^2+^ ions and CaC_2_O_4_ minerals, in contrast to the disordered peptide structure in bulk solution.

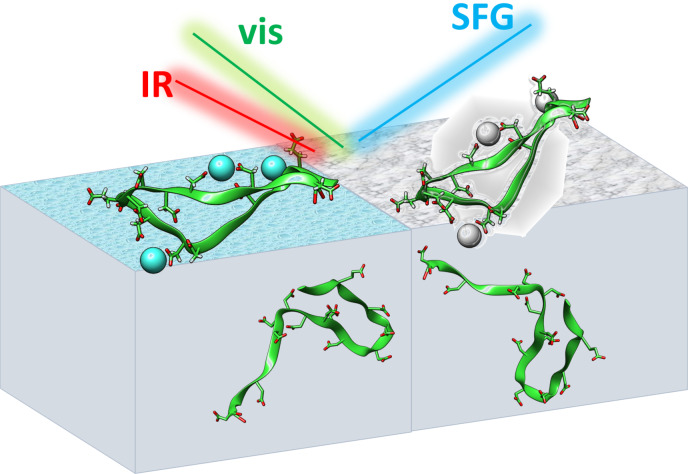

Calcium oxalate (CaC_2_O_4_) is one of the most common biominerals in nature. It also occurs as an abundant organic mineral found in sediments, hydrothermal vents, and plants.^[1]^ CaC_2_O_4_ has three hydrated phases: calcium oxalate monohydrate (COM), calcium oxalate dehydrate (COD), and calcium oxalate trihydrate (COT). Of these, COM is the thermodynamically most stable form, and constitutes the major mineral components of kidney stone^[2]^—a chronic human disease affecting 10 % of the population in developed countries. Due to the substantial pathological importance, different types of molecules have been applied for the inhibition of COM growth, including polyprotic acid,[^2d^, ^3^] polyacid polymers^[4]^ and peptides,[^2c^, ^2e^, ^4^, ^5^] and proteins.[^2d^, ^3a^, ^3b^, ^5b^, ^6^] Much effort has been aimed at elucidating the inhibition mechanism of specific molecular functional groups,[^3b^, ^3c^, ^3d^, ^7^] peptide sequence^[5d]^ and its modification,[^2c^, ^5c^] etc.

In the urinary system of the human body, a remarkably low concentration level of urinary proteins can effectively inhibit the growth of supersaturated CaC_2_O_4_ crystals, thereby avoiding larger stones.[^2c^, ^2d^, ^2e^, ^3b^, ^8^] Understanding the mechanism underlying the biological control of those urinary proteins over CaC_2_O_4_ mineralization is essential for developing novel therapies for kidney stone treatment. The most potent stone‐inhibiting protein in urine is osteopontin (OPN). OPN features peptide domains with acidic serine and aspartate‐rich motif (ASARM).^[9]^ Previous studies in bulk solutions have shown that ASARM peptides derived from OPN are capable of inhibiting CaC_2_O_4_ mineralization in vitro.[^2c^, ^2e^, ^10^] Hoyer et al. have found that the ASARM peptides derived from the sequence 62–85 of human OPN inhibit the growth of COM crystals regardless of the phosphorylation of the serine amino acids.^[2c]^ The authors further conclude potent inhibition requires specific charge density distribution. A similar conclusion was reached by Clark et al. in a study of the binding of protein G charge mutants to COM crystals. This study showed that the distribution of binding carboxylate groups from acidic side chains determines the binding site and orientation of the adsorbed protein.^[11]^ Clearly, direct interactions between OPN and oxalate mineral surfaces are quintessential,[^2c^, ^2d^, ^3b^, ^11^] yet the molecular structure and interaction of OPN sequences specifically at the interfaces relevant for CaC_2_O_4_ mineralization remain missing. Here, we show that, owing to the surface‐activity of OPN, the water–air interface can be ideally used to study the OPN/Ca^2+^ binding interface as well as OPN/CaC_2_O_4_ composite interface. While both the distribution of charge density and binding residues are affected by the folding structure of OPN protein,^[12]^ the question arises: how is OPN folded at the above active interfaces for CaC_2_O_4_ mineralization?

OPN protein is an intrinsically disordered protein.^[13]^ The ASARM OPN peptides are also disordered and lack a defined (e.g., α‐helical or β‐sheet) secondary structure.[^5c^, ^14^] However, the open and flexible motif for OPN allows for strong Ca^2+^ binding, which is expected to affect CaC_2_O_4_ mineralization. To determine the structure of OPN in contact with Ca^2+^ ions, and the molecular interactions involved in CaC_2_O_4_ mineralization, we probe the OPN‐derived peptides at the interfaces with Ca^2+^ binding and within CaC_2_O_4_ composite using surface‐specific vibrational sum‐frequency generation (SFG) spectroscopy. SFG has been successfully applied to probe different molecules (e.g., proteins, peptides, and water) at various interfaces.^[15]^ The SFG experiment relies on the frequency mixing of infrared (IR) and visible laser pulses. Molecular resonances excited by the IR pulse enhance the signal and yield a vibrational spectrum of interfacial molecules. The selection rules dictate that SFG signals can only be generated from ordered interfacial molecules for which, on average, centrosymmetry is broken.^[16]^ As such, SFG is well suitable to probe the structure and interaction of OPN molecules, specifically at the interface and *in situ*. Here, we investigate a representative ASARM peptide derived from region 62–85 of human OPN (H_2_NSNESHDHMDDMDDEDDDDHVDSQDCOOH), abbreviated as OPN peptide below.

Before focusing OPN peptides at the interface, we first perform CD measurements (180–260 nm) to determine the secondary structure content of unbound OPN peptides in bulk water and potential changes when adding Ca^2+^ and subsequently C_2_O_4_^2−^ for CaC_2_O_4_ mineralization. As seen in Figure 1 a, the CD spectra of OPN peptides all exhibit one dominant negative ellipticity at 200 nm and a small secondary band at 230 nm. These two bands correspond to the π–π* and n–π* transition of the amide bond, respectively.^[17]^ In agreement with studies on disordered oligopeptides, the signatures reflect a major contribution from random coils and β‐turns,^[18]^ while lacking in defined secondary structures such as α‐helix or β‐sheet. The structure inferred from CD measurement is in good agreement with previous studies on OPN peptides.[^5c^, ^14^] Multivariate secondary structure analysis was performed using the partial least squares method fitted against standard proteins (i.e., lysozyme, cytochrome *c*) within the JASCO library.^[19]^ The secondary structures remain essentially unchanged upon adding Ca^2+^ and subsequently C_2_O_4_^2−^ ions. A secondary structure analysis shown in Figure 1 b reveals approximately 21 % α‐helix, 7 % β‐sheet, 25 % β‐turns, and 47 % random coils.

**Figure 1:**
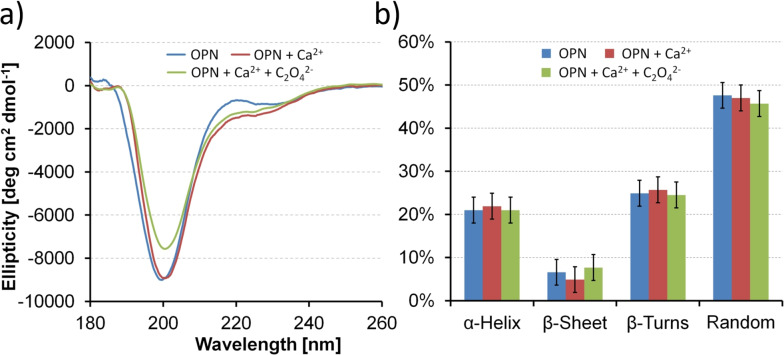
a) Circular dichroism spectra of OPN peptides in water solution (blue), with addition of Ca^2+^ cations (red) and subsequently C_2_O_4_^2−^ anions for CaC_2_O_4_ mineralization (green). b) Secondary structure estimation of the respective OPN peptides in water solution (blue), with addition of Ca^2+^ cations (red), and subsequently C_2_O_4_^2−^ anions (green).

The secondary structure for OPN peptides in solution does not necessarily represent their structure at the interface. Figure 2 illustrates our strategy tackling OPN structure at the interface during CaC_2_O_4_ mineralization: The peptides were firstly allowed to adsorb at the air–water interface (top panel). The peptides at the air–water interface provide an ideal two‐dimensional “soft” interface,^[20]^ allowing us to study the interfacial interaction of OPN peptides with Ca^2+^ ions and further CaC_2_O_4_ minerals. Adding Ca^2+^ cations is expected to bind and refold interfacial peptides (middle panel). The subsequent addition of C_2_O_4_^2−^ anions will initiate interfacial mineralization within refolded peptides (bottom panel). Surface pressure measurements show that the adsorbed peptides reach a surface pressure of 25 mN m^−1^. The surface pressure decreases only slightly with the addition of Ca^2+^ and C_2_O_4_^2−^ ions, suggesting the nearly constant density of interfacial peptides throughout the mineralization process (Figure S1).

**Figure 2:**
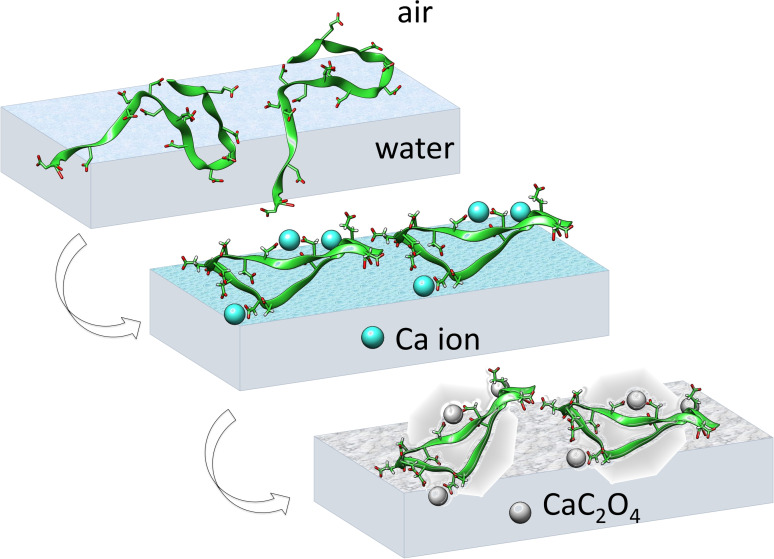
Scheme illustrating the OPN peptides at the air–solution interface (top), with Ca^2+^ ions (middle), and CaC_2_O_4_ minerals (bottom). Initially, OPN peptides are disordered at the air–water interface (top), but will refold into an ordered β‐turn structure upon interfacial binding to Ca^2+^ (middle) and CaC_2_O_4_ minerals (bottom). The OPN peptides show dynamic features in the solution during the simulation. The depicted structures are generated from simulation frames to illustrate the interaction‐induced ordering at the interface.

Interfacial mineralization is expected to complete within 20 minutes after injecting C_2_O_4_^2−^ anions.^[21]^ A thin nanosheet consisting of CaC_2_O_4_ minerals and OPN peptides can be lifted off the interface with a transmission electron microscopy (TEM) grid using the Langmuir–Schaefer approach.^[21a]^ CaC_2_O_4_ mineralization by peptides in solution has been extensively reported, while interfacial mineralization, resulting in a peptide/CaC_2_O_4_ composite, has not been well studied.^[21a]^ We applied TEM and X‐ray photoelectron spectroscopy (XPS) to characterize the obtained interfacial nanosheet composites. Figure 3 a shows the TEM image of the nanosheet, intact over micrometers. The obtained thin peptide–oxalate nanosheet provides an ideal platform to study interfacial CaC_2_O_4_ mineralization by OPN peptides. The chemical composition of the composite nanosheet was confirmed by XPS: distinct Ca 2p and N 1s emission peaks (Figure S2) indicate the presence of peptides and oxalate minerals. The high‐resolution TEM image in Figure 3 b highlights the nanocrystalline CaC_2_O_4_ particles within the sheet. The obtained nanocrystals indicate that OPN peptides are highly efficient nucleators for interfacial mineralization. The exact crystal composition (e.g., COM or COD) could not be well determined from diffraction measurements, which may be caused by the crystal decomposition as induced by the high‐energy electron beam.^[22]^ We further examine the CaC_2_O_4_ crystals by OPN peptides in bulk solution; the tetragonal morphology and diffraction analysis indicate COD crystals are obtained (Figure S3).

**Figure 3:**
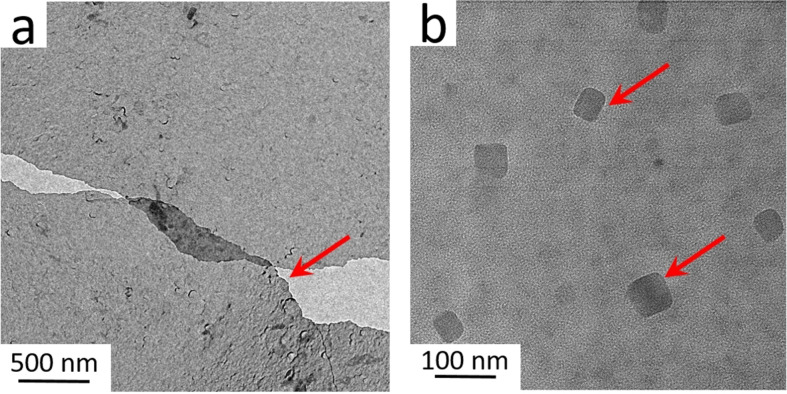
a) TEM image showing the nanosheet composed of OPN peptides and CaC_2_O_4_ minerals. The red arrow marks the raptured film. b) High‐resolution TEM image of the crystalline CaC_2_O_4_ mineral nanocrystals within the nanosheet. The red arrows highlight CaC_2_O_4_ crystals.

To glean molecular‐level insights into OPN peptides when mineralizing an interface, we applied surface‐specific SFG spectroscopy. SFG selectively probes the outermost approximately 2 nm of the water surface, allowing to probe interfacial peptides without contribution from peptide molecules in bulk solution. Figure 4 a shows SFG spectra in the CH/OH region for OPN peptides adsorbed at the air–water interface, with Ca^2+^ ion interaction and further CaC_2_O_4_ mineralization. The CH bands appear at 2878 cm^−1^ and 2950 cm^−1^, corresponding to the symmetric CH_3_ stretch, and a combination of CH_3_ Fermi resonance and CH_2_ stretch from different amino acid side chains, respectively.^[23]^ The water response gives rise to two broad OH stretch bands centered at 3241 cm^−1^ and 3410 cm^−1^, with an overall first moment centered at 3276 cm^−1^, indicating the presence of strongly and weakly hydrogen‐bonded water molecules.[^15c^, ^24^] The OH response is rather intense, owing to the negatively charged peptides, which align the water dipoles and thereby break centrosymmetry within the interfacial water layers. The OH response decreases with adding Ca^2+^ ions, implying decreased water alignment; the first moment value increases by 63 cm^−1^ (from 3276 to 3339 cm^−1^), suggesting weakening of the hydrogen bond strength. The observed change in OH bands is mainly due to Ca^2+^ ions, which interact at the OPN interface and screen the peptide charge, thereby modifying the structure of interfacial water. No obvious spectral change is observed during CaC_2_O_4_ mineralization.

**Figure 4:**
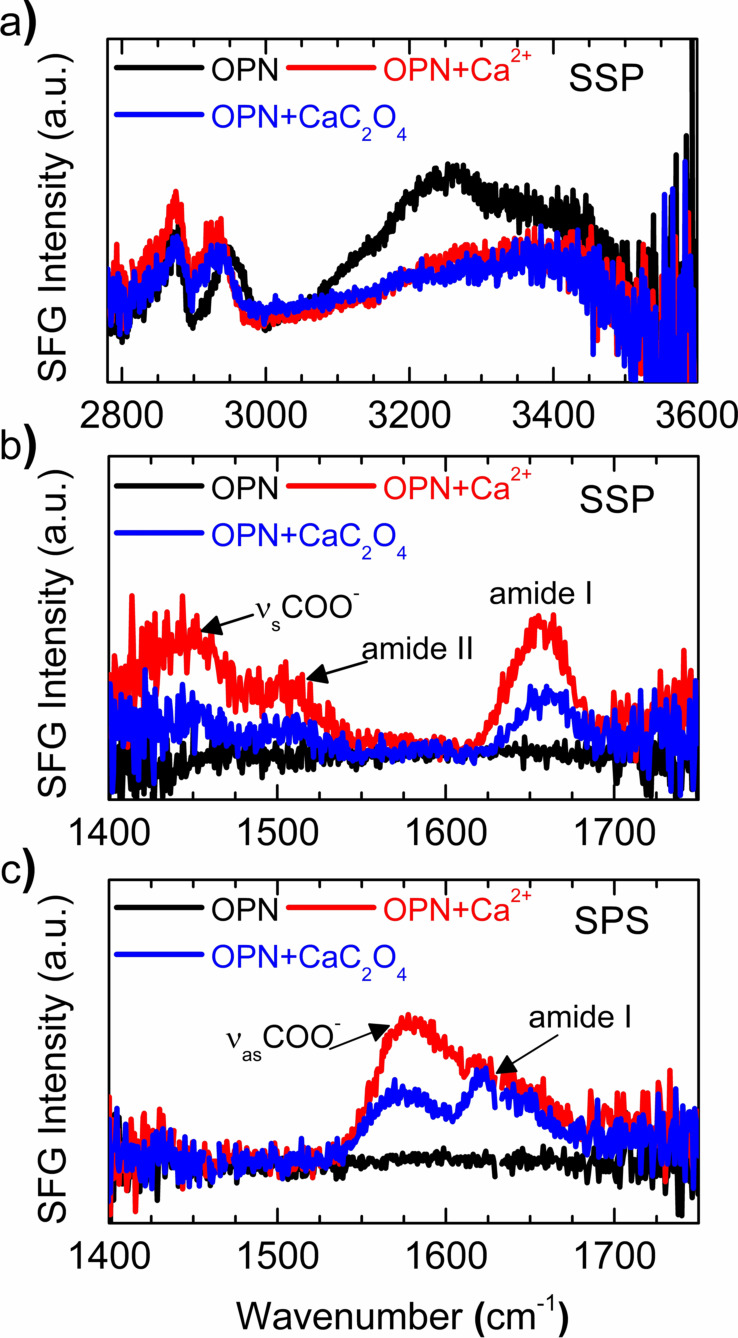
SFG spectra in CH/OH (a) and amide I region (b,c) for OPN peptides adsorbed at the air–water interface (black), with Ca^2+^ ion interaction (red), and further CaC_2_O_4_ mineralization (blue). The spectra were acquired under SSP (a,b) and SPS (c) polarization combinations.

The substantial perturbation of the peptide hydration shell prompts us to look closely at the interfacial peptides. To this end, we record SFG spectra in the amide I region, for SSP (S‐polarized SFG and Vis, P‐polarized IR) and SPS (S‐polarized SFG, P‐polarized Vis, S‐polarized IR) polarization combinations, as shown in Figure 4 b,c. Both spectra for original OPN peptides show nearly zero signal, suggesting a complete lack of peptide order, consistent with the intrinsically disordered motif of OPN in bulk solution from CD measurement. When adding Ca^2+^ ions, the SSP spectra exhibit three distinct SFG bands: one side chain band at circa 1447 cm^−1^, attributed to symmetric COO^−^ stretch from the deprotonated acidic side‐chains (e.g. of glutamic and aspartic acid); and two backbone bands at approximately 1507 cm^−1^ and 1652 cm^−1^, assigned to amide II and amide I bands, respectively. Similarly, the SPS spectra in Figure 4 c reveal two dominating bands for peptides with Ca^2+^: one at approximately 1570 cm^−1^ from the asymmetric COO^−^ stretch of the deprotonated acidic side‐chains and another at approximately 1611 cm^−1^ for backbone amide I mode. Apparently, as sketched in Figure 2, the binding of Ca^2+^ ions restructures the peptide backbone and binding side chains from a disordered into a well‐ordered motif. Following interfacial CaC_2_O_4_ mineralization, this order diminishes somewhat, as revealed by the reduced intensities of the SFG peaks.

The resonance frequency of the side‐chain mode is sensitive to the COO−Ca binding geometry, and the observed frequency of circa 1570 cm^−1^ reveals bidentate binding.^[21a]^ The well‐coordinated Ca^2+^ ions refold the disordered backbone into a defined secondary structure, as evidenced by the distinct amide I peaks. The fitting frequencies at 1652 cm^−1^ in SSP spectra and 1611 cm^−1^ in SPS spectra (Table S1 and S2), together with comparing analogous spectra for leucine–glutamic acid sequences (Figure S5), suggest the β‐turn secondary structure,[^12a^, ^21a^, ^25^] which is in agreement with previous simulation studies for OPN sequences binding at the CaC_2_O_4_ mineral surface.[^14^, ^26^] Interestingly, the amide II band is also well discernible in the SSP spectra (Figure 4 b). The amide II mode arises from the out‐of‐phase combination of the C−N stretch and the N−H in‐plane deformation.^[27]^ It is Raman inactive,^[28]^ and, therefore, in principle, not SFG‐detectable since the SFG signal is proportional to a tensor product of the IR transition dipole moment and the Raman polarizability.^[16]^ However, recent studies have shown that amide II modes can be visible in SFG spectra for folding motifs with extended tertiary structure (e.g. multi‐stranded β‐sheets), where the long‐range vibrational coupling from intermolecular β‐sheet contacts can enhance the amide II signals.^[29]^ Here the observable amide II band suggests the assembly of well‐aligned β‐turn peptides, as caused by the intercalating Ca^2+^ ions. The ordered peptide assemblies may act as a critical structural intermediate that guides interfacial mineralization, resulting in 2D‐nanosheet‐containing crystalline minerals (Figure 3). The assembled peptides can also be fingerprinted in high‐resolution TEM imaging (Figure S4). During mineralization, the consumption of coordinated Ca^2+^ ions by C_2_O_4_^2−^ breaks the bidentate binding and results in the decreased order in both backbone and side chains. Interestingly, the concentration of OPN peptides in solution is 17.7 nm, which is below physiological conditions; adding Ca^2+^ and subsequent C_2_O_4_^2−^ ions in a higher mm range will not change the structure of free peptides in solution, but rather refold interfacial peptides into a well‐defined β‐turn motif.

In conclusion, we have applied surface‐specific SFG spectroscopy to probe the structure of the central OPN fragment, its interaction with Ca^2+^, and CaC_2_O_4_ mineralization. Contrary to the unchanged disordered structure in bulk solution, we show that the bidentate interaction with Ca^2+^ ions refolds the OPN peptides into a well‐ordered and assembled β‐turn motif at the OPN‐covered aqueous Ca^2+^ solution surface, where interfacial mineralization can subsequently occur. Our results highlight the significantly different behavior of OPN peptides at the interface and in bulk solution for CaC_2_O_4_ mineralization. In particular, the critical peptide–Ca^2+^ binding interface should be taken into account in CaC_2_O_4_ mineralization, for example, for the treatment of kidney stones; such interfacial processes may also be relevant for calcium phosphate biomineralization in bone formation.

## Conflict of interest

The authors declare no conflict of interest.

## Supporting information

As a service to our authors and readers, this journal provides supporting information supplied by the authors. Such materials are peer reviewed and may be re‐organized for online delivery, but are not copy‐edited or typeset. Technical support issues arising from supporting information (other than missing files) should be addressed to the authors.

Supporting Information

## References

[1] T. Echigo, M. Kimata, Can. Mineral. 2010, 48, 1329–1357.

[2a] R. L. Ryall, World J. Urol. 1997, 15, 155–164; 9228722 10.1007/BF02201852

[2b] F. L. Coe, A. Evan, E. Worcester, J. Clin. Invest. 2005, 115, 2598–2608; 16200192 10.1172/JCI26662 PMC1236703

[2c] J. R. Hoyer, J. R. Asplin, L. Otvos, Kidney Int. 2001, 60, 77–82; 11422738 10.1046/j.1523-1755.2001.00772.x

[2d] S. R. Qiu, A. Wierzbicki, C. A. Orme, A. M. Cody, J. R. Hoyer, G. H. Nancollas, S. Zepeda, J. J. De Yoreo, Proc. Natl. Acad. Sci. USA 2004, 101, 1811–1815; 14766970 10.1073/pnas.0307900100 PMC357009

[2e] Y. C. Chien, D. L. Masica, J. J. Gray, S. Nguyen, H. Vali, M. D. Mckee, J. Biol. Chem. 2009, 284, 23491–23501. 19581305 10.1074/jbc.M109.021899 PMC2749123

[3a] J. J. De Yoreo, S. R. Qiu, J. R. Hoyer, Am. J. Physiol. Renal Fluid Electrolyte Physiol. 2006, 291, F1123–F1131; 17082348 10.1152/ajprenal.00136.2006

[3b] X. X. Sheng, T. S. Jung, J. A. Wesson, M. D. Ward, Proc. Natl. Acad. Sci. USA 2005, 102, 267–272; 15625112 10.1073/pnas.0406835101 PMC544292

[3c] J. Chung, R. Sosa, J. D. Rimer, Cryst. Growth Des. 2017, 17, 4280–4288;

[3d] J. Chung, M. G. Taylor, I. Granja, J. R. Asplin, G. Mpourmpakis, J. D. Rimer, Cryst. Growth Des. 2018, 18, 5617–5627.

[4] T. Jung, X. X. Sheng, C. K. Choi, W. S. Kim, J. A. Wesson, M. D. Ward, Langmuir 2004, 20, 8587–8596. 15379479 10.1021/la0488755

[5a] S. W. Guo, M. D. Ward, J. A. Wesson, Langmuir 2002, 18, 4284–4291;

[5b] A. A. Campbell, A. Ebrahimpour, L. Perez, S. A. Smesko, G. H. Nancollas, Calcif. Tissue Int. 1989, 45, 122–128; 2476205 10.1007/BF02561411

[5c] L. J. Wang, X. Y. Guan, R. K. Tang, J. R. Hoyer, A. Wierzbicki, J. J. De Yoreo, G. H. Nancollas, J. Phys. Chem. B 2008, 112, 9151–9157; 18611047 10.1021/jp804282u PMC2743538

[5d] L. J. Wang, S. R. Qiu, W. Zachowicz, X. Y. Guan, J. J. DeYoreo, G. H. Nancollas, J. R. Hoyer, Langmuir 2006, 22, 7279–7285. 16893227 10.1021/la060897z

[6a] S. Farmanesh, S. Ramamoorthy, J. H. Chung, J. R. Asplin, P. Karande, J. D. Rimer, J. Am. Chem. Soc. 2014, 136, 367–376; 24313314 10.1021/ja410623q

[6b] S. Farmanesh, J. H. Chung, R. D. Sosa, J. H. Kwak, P. Karande, J. D. Rimer, J. Am. Chem. Soc. 2014, 136, 12648–12657. 25119124 10.1021/ja505402r

[7] X. X. Sheng, M. D. Ward, J. A. Wesson, J. Am. Chem. Soc. 2003, 125, 2854–2855. 12617634 10.1021/ja029575h

[8] B. Grohe, J. O'Young, D. A. Ionescu, G. Lajoie, K. A. Rogers, M. Karttunen, H. A. Goldberg, G. K. Hunter, J. Am. Chem. Soc. 2007, 129, 14946–14951. 17994739 10.1021/ja0745613

[9] S. G. Liu, P. S. N. Rowe, L. Vierthaler, J. P. Zhou, L. D. Quarles, J. Endocrinol. 2007, 192, 261–267. 17210763 10.1677/joe.1.07059 PMC3357085

[10] Y. Liu, H. Y. Mao, X. F. Liu, L. J. Qiao, R. Guo, CrystEngComm 2014, 16, 8841–8851.

[11] R. H. Clark, A. A. Campbell, L. A. Klumb, C. J. Long, P. S. Stayton, Calcif. Tissue Int. 1999, 64, 516–521. 10341024 10.1007/s002239900642

[12a] H. Lu, H. Lutz, S. J. Roeters, M. A. Hood, A. Schafer, R. Munoz-Espi, R. Berger, M. Bonn, T. Weidner, J. Am. Chem. Soc. 2018, 140, 2793–2796; 29420020 10.1021/jacs.8b00281

[12b] J. E. Baio, A. Zane, V. Jaeger, A. M. Roehrich, H. Lutz, J. Pfaendtner, G. P. Drobny, T. Weidner, J. Am. Chem. Soc. 2014, 136, 15134–15137. 25285787 10.1021/ja5078238 PMC4608251

[13] D. Kurzbach, G. Platzer, T. C. Schwarz, M. A. Henen, R. Konrat, D. Hinderberger, Biochemistry 2013, 52, 5167–5175. 23848319 10.1021/bi400502c PMC3737600

[14] P. V. Azzopardi, J. O'Young, G. Lajoie, M. Karttunen, H. A. Goldberg, G. K. Hunter, PloS One 2010, 5, e9330. 20174473 10.1371/journal.pone.0009330 PMC2824833

[15a] S. Roy, P. A. Covert, W. R. FitzGerald, D. K. Hore, Chem. Rev. 2014, 114, 8388–8415; 24405207 10.1021/cr400418b

[15b] B. Ding, J. Jasensky, Y. Li, Z. Chen, Acc. Chem. Res. 2016, 49, 1149–1157; 27188920 10.1021/acs.accounts.6b00091

[15c] M. Bonn, Y. Nagata, E. H. G. Backus, Angew. Chem. Int. Ed. 2015, 54, 5560–5576; 10.1002/anie.201411188 25877765

[16a] Y. R. Shen, The Principles of Nonlinear Optics, Wiley, New York, 1984;

[16b] A. G. Lambert, P. B. Davies, D. J. Neivandt, Appl. Spectrosc. Rev. 2005, 40, 103–145.

[17] C. A. Bush, S. K. Sarkar, K. D. Kopple, Biochemistry 1978, 17, 4951–4954. 82446 10.1021/bi00616a015

[18a] P. F. J. Fuchs, A. M. J. J. Bonvin, B. Bochicchio, A. Pepe, A. J. P. Alix, A. M. Tamburro, Biophys. J. 2006, 90, 2745–2759; 16443656 10.1529/biophysj.105.074401 PMC1414573

[18b] L. B. Chemes, L. G. Alonso, M. G. Noval, G. de Prat-Gay, Methods Mol. Biol. 2012, 895, 387–404. 22760329 10.1007/978-1-61779-927-3_22

[19] W. C. Johnson, Proteins Struct. Funct. Bioinf. 1999, 35, 307–312.

[20a] D. C. Popescu, M. M. J. Smulders, B. P. Pichon, N. Chebotareva, S. Y. Kwak, O. L. J. van Asselen, R. P. Sijbesma, E. DiMasi, N. A. J. M. Sommerdijk, J. Am. Chem. Soc. 2007, 129, 14058–14067; 17944471 10.1021/ja075875t

[20b] S. Cavalli, D. C. Popescu, E. E. Tellers, M. R. J. Vos, B. P. Pichon, M. Overhand, H. Rapaport, N. A. J. M. Sommerdijk, A. Kros, Angew. Chem. Int. Ed. 2006, 45, 739–744; 10.1002/anie.200501654 16231381

[21a] H. Lu, A. Schafer, H. Lutz, S. J. Roeters, I. Lieberwirth, R. Munoz-Espi, M. A. Hood, M. Bonn, T. Weidner, J. Phys. Chem. Lett. 2019, 10, 2170–2174; 30978286 10.1021/acs.jpclett.9b00684 PMC6727606

[21b] V. Fischer, K. Landfester, R. Muñoz-Espí, Cryst. Growth Des. 2011, 11, 1880–1890.

[22] E. Ruiz-Agudo, A. Burgos-Cara, C. Ruiz-Agudo, A. Ibanez-Velasco, H. Colfen, C. Rodriguez-Navarro, Nat. Commun. 2017, 8, 768. 28974672 10.1038/s41467-017-00756-5 PMC5626694

[23a] M. R. Watry, G. L. Richmond, J. Phys. Chem. B 2002, 106, 12517–12523;

[23b] G. J. Holinga, R. L. York, R. M. Onorato, C. M. Thompson, N. E. Webb, A. P. Yoon, G. A. Somorjai, J. Am. Chem. Soc. 2011, 133, 6243–6253. 21452815 10.1021/ja1101954

[24] Q. Du, E. Freysz, Y. R. Shen, Science 1994, 264, 826–828. 17794723 10.1126/science.264.5160.826

[25a] K. T. Nguyen, J. T. King, Z. Chen, J. Phys. Chem. B 2010, 114, 8291–8300; 20504035 10.1021/jp102343h PMC2896324

[25b] B. R. Singh, Infrared Analysis of Peptides and Proteins: Principles and Applications, American Chemical Society, Washington, DC, 2000.

[26] G. K. Hunter, J. O'Young, B. Grohe, M. Karttunen, H. A. Goldberg, Langmuir 2010, 26, 18639–18646. 20527831 10.1021/la100401r

[27] A. Barth, C. Zscherp, Q. Rev. Biophys. 2002, 35, 369–430. 12621861 10.1017/s0033583502003815

[28] S. Krimm, J. Bandekar, Adv. Protein Chem. 1986, 38, 181–364. 3541539 10.1016/s0065-3233(08)60528-8

[29a] J. J. Tan, J. H. Zhang, Y. Luo, S. J. Ye, J. Am. Chem. Soc. 2019, 141, 1941–1948; 30621387 10.1021/jacs.8b08537

[29b] L. Fu, D. Q. Xiao, Z. G. Wang, V. S. Batista, E. C. Y. Yan, J. Am. Chem. Soc. 2013, 135, 3592–3598. 23394622 10.1021/ja3119527 PMC9208335

