# Supplementary material for: Intrinsically Disordered Osteopontin Fragment Orders During Interfacial Calcium Oxalate Mineralization
Source: Angew Chem Int Ed Engl. 2021 Jul 16;60(34):18577–81. doi: 10.1002/anie.202105768 (PMC8457088; doi:10.1002/anie.202105768)
Supplement: Supplementary file 1 — Supporting Information [file ANIE-60-18577-s001.pdf]

Angewandte Chemie
Eine Zeitschrift der Gesellschaft Deutscher Chemiker

Supporting Information

Intrinsically Disordered Osteopontin Fragment Orders During Interfacial Calcium Oxalate Mineralization

Hao Lu, David Yuen Wah Ng, Ingo Lieberwirth, Tobias Weidner, and Mischa Bonn**

anie_202105768_sm_miscellaneous_information.pdf

Supporting Information

Table of Content:

1. Experimental Section S2
2. Surface Pressure S3
3. X-ray Photoelectron Spectroscopy (XPS) S4
4. Transmission Electron Microscopy (TEM) S5-S6
5. Circular Dichroism (CD) S6
6. Vibrational Sum Frequency Generation (SFG) Spectroscopy S7-S9
7. References S9

1. Experimental Section

Osteopontin 62-85 peptides with sequence H_2NSNESHDHMDDMDDEDDDDHVDSQDCOOH were purchased from GenScript.

1 mg OPN peptides were dissolved into 1 mL MilliQ (D_2O was used as a solvent for SFG measurement in the amide I region) and injected into the subphase in the trough, where the solutions are mixed with 18 mL MilliQ, 0.19 mL KCl (3 M/L, final concentration 28.5 mM/L), and 0.78 mL KOH (0.1 M/L, final concentration 3.9 mM/L). The final concentration of OPN peptide is 17.7 nM (nanoMolar), which is below physiological concentration. The pH of the final solution is 10.87, and the acidic amino acids of OPN peptides are fully deprotonated at this pH. The solution in the trough was stabilized for 1 hour, ensuring the equilibrium of peptide adsorption at the air-water interface. 0.78 mL $CaCl_2$ (0.1 M/L) was injected into the subphase of peptide solution, the injected Ca^{2+} ions are coordinated at the charged peptide interface; after 2 hours for Ca^{2+} ion interaction, the calcium oxalate (CaC_2O_4) mineralization was initiated by injecting 0.78 mL (0.1 M/L) $Na_2C_2O_4$ into the sub-phase of solution. After 20 minutes of mineralization, the fabricated OPN peptide-CaC_2O_4 films were lifted off the interface using the Langmuir-Schaefer technique using copper transmission electron microscopy (TEM) grids.

The CaC_2O_4 mineralized peptide films were characterized by X-ray photoelectron spectroscopy (XPS) and TEM.

2. Surface Pressure measurements

Surface pressure has been measured using a Langmuir tensiometer (Kibron, Finland). The Teflon trough was thoroughly cleaned sequentially with acetone, ethanol, and milliQ water, and dried under a nitrogen stream prior to measurements. The surface pressure (π) was normalized with pure water to 0 mN/m. The SFG spectra were collected when surface pressure reached equilibrium.

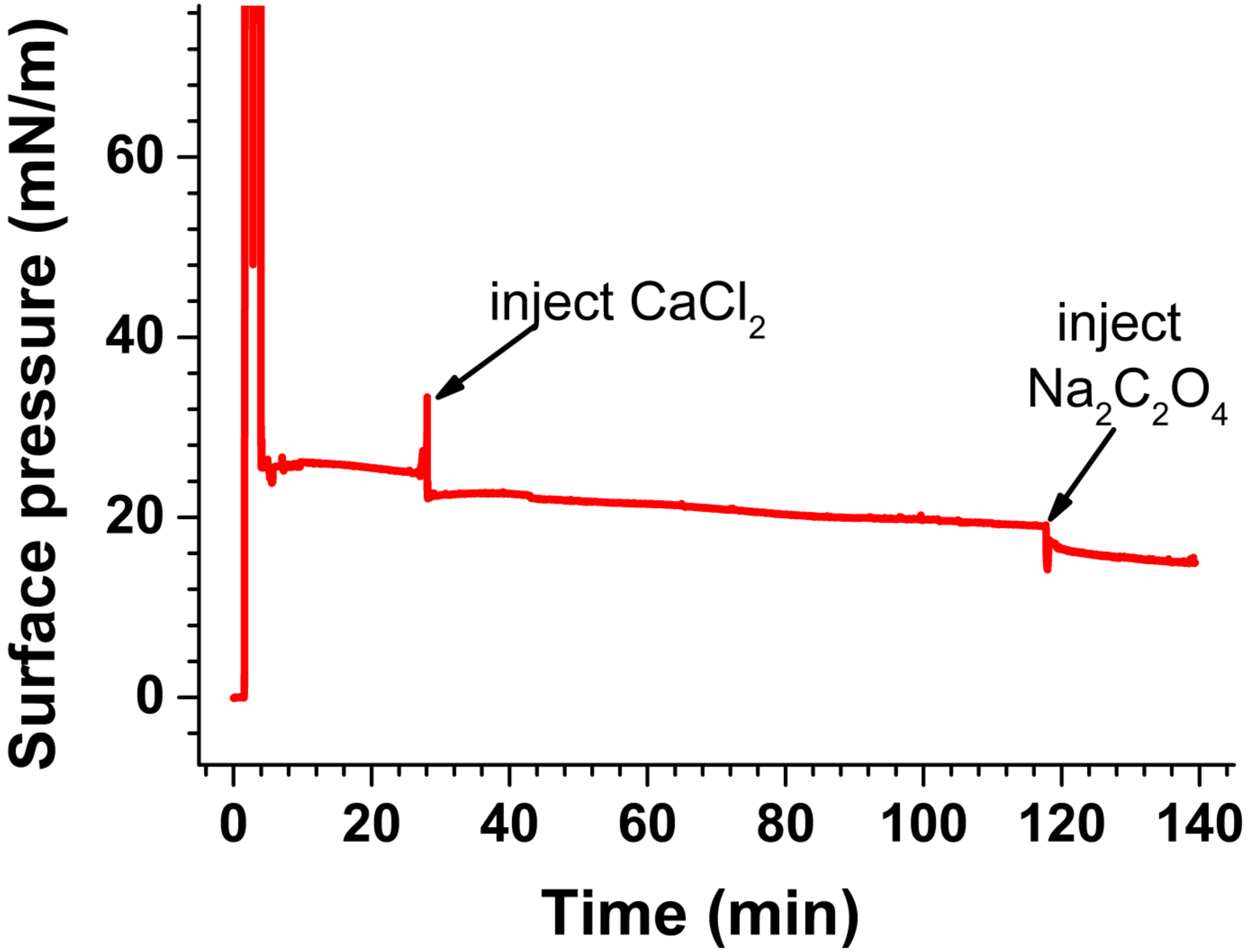

Figure S1. Surface pressure of the OPN peptides assembled at the air-water interface, and with subsequent injection of $CaCl_2$, and $Na_2C_2O_4$ for CaC_2O_4 mineralization at the interface.

3. XPS

X-ray Photoelectron Spectroscopy (XPS) was conducted using a Kratos Axis Ultra^{DLD} spectrometer (Kratos, Manchester, England) using an Al Kα excitation source with a photon energy of 1487 eV. The data was acquired in the hybrid mode using a 0° take-off angle, defined as the angle between the surface normal and the axis of the analyzer lens. XP spectra were collected with setting analyzer pass energy at 80 eV. A linear background was subtracted for all peak quantifications. The peak areas were normalized by the manufacturer-supplied sensitivity factors and surface concentrations were calculated using CasaXPS software. Neutralizer was always used during spectra collection. Binding energy scales were calibrated to C 1s emission at 284.8 eV.[1]

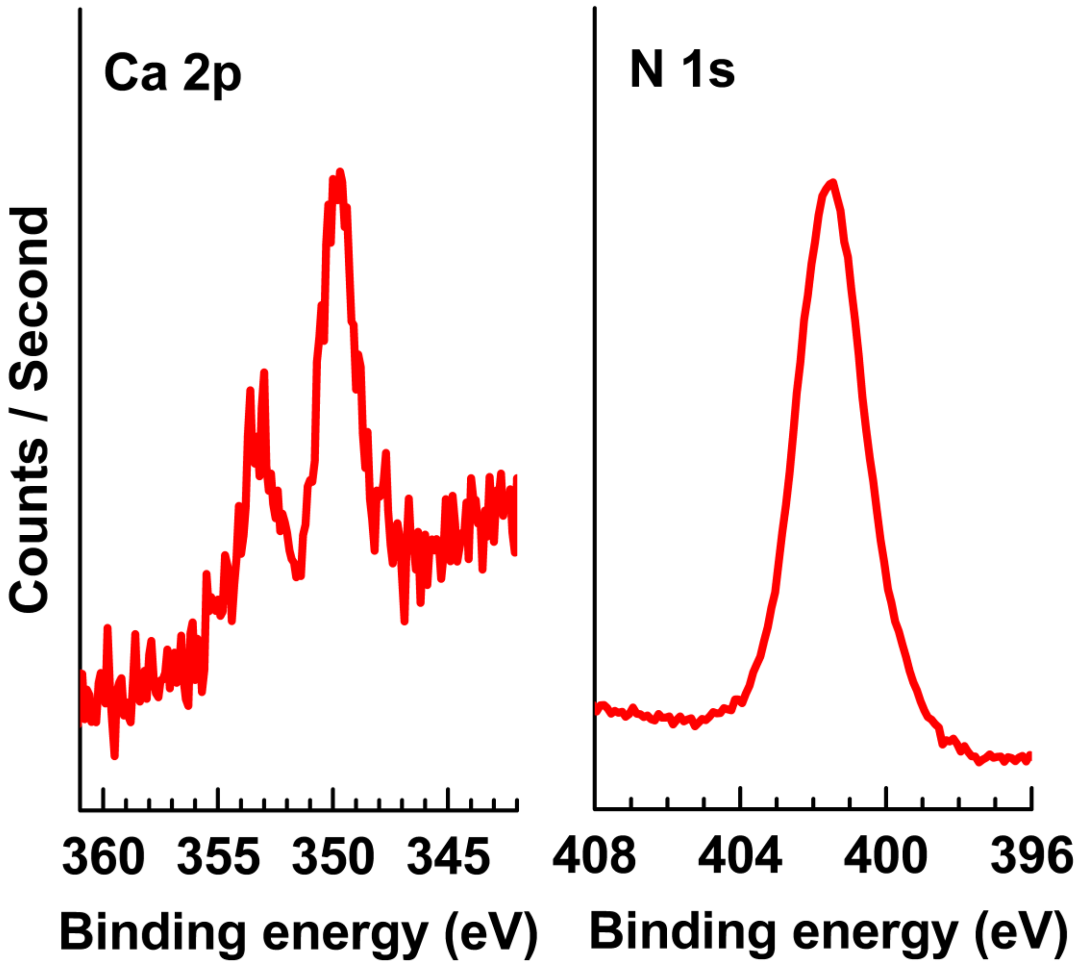

Figure S2. Ca 2p and N 1s XP spectra of the OPN-CaC_2O_4 composite nanosheet.

4. TEM

TEM examination was done using a FEI Tecnai F20 operated at an acceleration voltage of 200 kV. Micrographs were recorded on a Gatan Ultrascan CCD Camera.

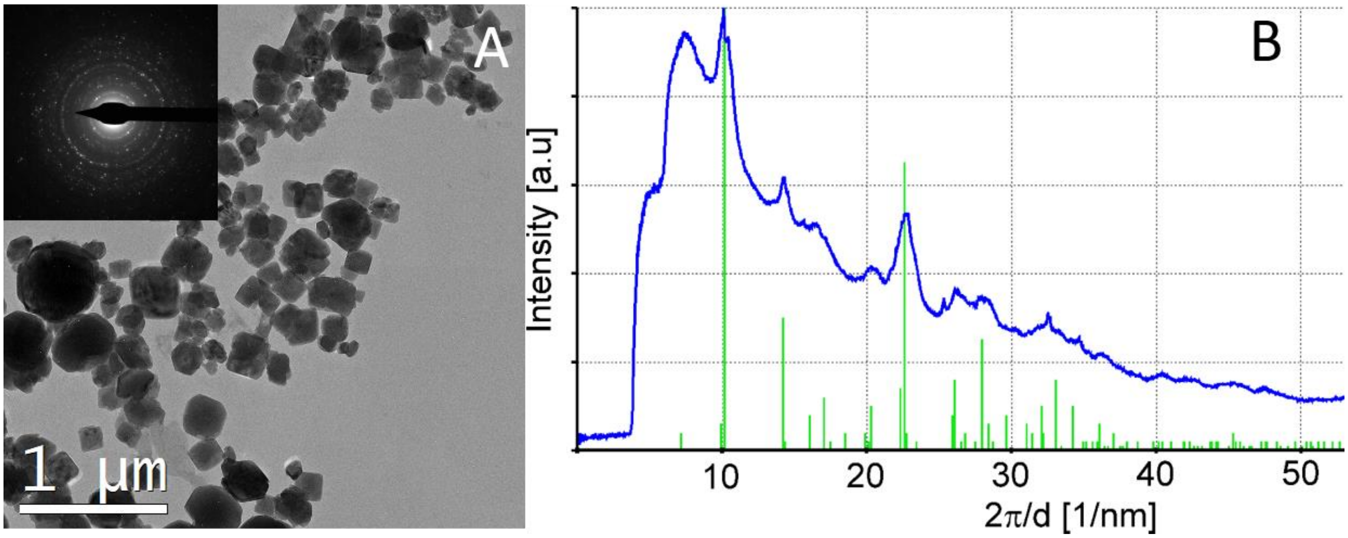

Figure S3. (A) TEM micrograph and corresponding diffraction pattern (inset) show the crystalline tetragonal calcium oxalate dehydrate (COD) crystals obtained by OPN peptides in bulk solution. **(B)** The diffractogram displays the radial intensity profile of the diffraction pattern (blue) together with the index peaks (green) for Weddellite (COD) as extracted from the powder diffraction database (pdf2) no 17-0541. From the match of the peaks with those of the diffractogram, the crystal structure is identified to be Weddellite (COD).

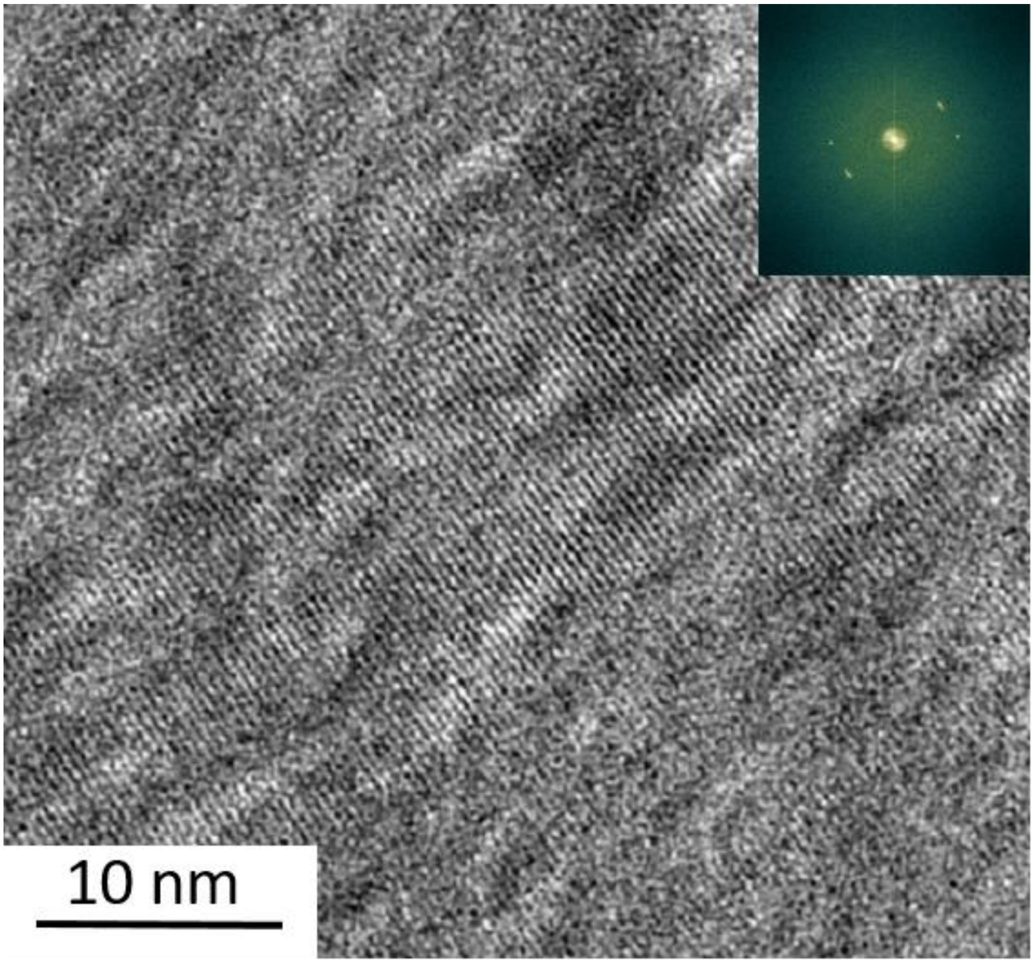

Figure S4. High-resolution TEM image visualizing the ordered OPN peptides within the peptide-oxalate composite nanosheet.

5. Circular Dichroism

Circular dichroism spectroscopy was recorded on a JASCO J-1500 spectrophotometer at 25°C using a 0.1 cm path length quartz cuvette from Hellma Analytical (Mülheim, Germany). Data points were collected at a resolution of 0.2 nm, an integration time of 2 s and a scanning speed of 5 nm min^{-1}. Each spectrum was the result of three accumulated independent scans. Background subtraction was accomplished using the solvent.

The concentration of peptide in the measurement was diluted to 0.25 mg mL^{-1} (2.13 mM) to achieve the optimum high tension (HT) voltage.

6. Vibrational Sum Frequency Generation (SFG) Spectroscopy:

The vibrational SFG spectra were obtained by overlapping, in time and space, the visible and IR pulses. A Ti:Sapphire amplified system (Spitfire Ace, Spectra Physics Inc.) delivers 35 fs long pulses at a central wavelength of ~800 nm and 1 KHz repetition rate. The beam is split in two parts: one it is spectrally narrowed using a Fabry-Perot etalon to achieve spectral resolution of 15cm^{-1} (lambda=800, E~25mJ/pulse). The other part is used to generate tunable broadband IR pulses thanks to a parametric optical amplifier followed by a noncollinear difference frequency generation module (TOPAS Prime, LightConversion). The average power is 2μJ/pulse at a wavelength of 6000 nm and 3μJ/pulse at a wave-length of 3000 nm. Visible and IR beams are focused onto the sample using respectively a 20cm and 5cm focal length (FL) lenses. The polarization of both beams can be controlled (s or p) with a polarizer and a half waveplate. Beams are temporally and spatially overlapped at the sample position. The SFG signal is generated with visible and IR beam angles of 55° and 60° respective to the surface normal, and is colli-mated using a 20cm FL lens, further focused into a spectrograph using a 5cm FL achromatic lens, dis-persed by a grating and collected by an intensified CCD camera. The polarization of the SFG signal can be well controlled like Visible and IR beams.

Each spectrum was acquired for 10 minutes, and the spectra are normalized by non-resonance reference spectra of z-cut quartz crystal after background correction. Spectra in amide I region were recorded in the SSP (sum, visible, and infrared) and SPS polarization combination, and spectra were calibrated according to the vibration bands of water vapor. For SFG experiments in amide I region, D_2O solvent was used to avoid the spectra interference from the bending mode of H_2O. Spectra in CH/OH region were recorded in SSP polarization combinations, and referenced by the absorption bands of polystyrene.

SFG spectra were fitted by Lorentzian peak shapes according to the following equation:

$$I_{SFG} \propto \left|\chi^{(2)}\right|^2 = \left|\chi_{NR}^{(2)} + \chi_R^{(2)}\right|^2 = \left|A_{NR}e^{i\Phi_{NR}} + \sum_{n=1}^{\infty} \frac{A_n}{\omega_{IR} - \omega_n - i\Gamma_n}\right|^2 \tag{1}$$

In equation (1) above, the susceptibility $\chi^{(2)}$ consists of a non-resonant ($\chi_{NR}^{(2)}$) and a resonant ($\chi_R^{(2)}$) term. A_{NR} and ϕ_{NR} are the amplitude and phase of non-resonant signal, respectively. A_n is the amplitude of resonant signal, ω_n is the resonant frequency, ω_{IR} is the infrared frequency, and Γ_n is the width of transi-tion.

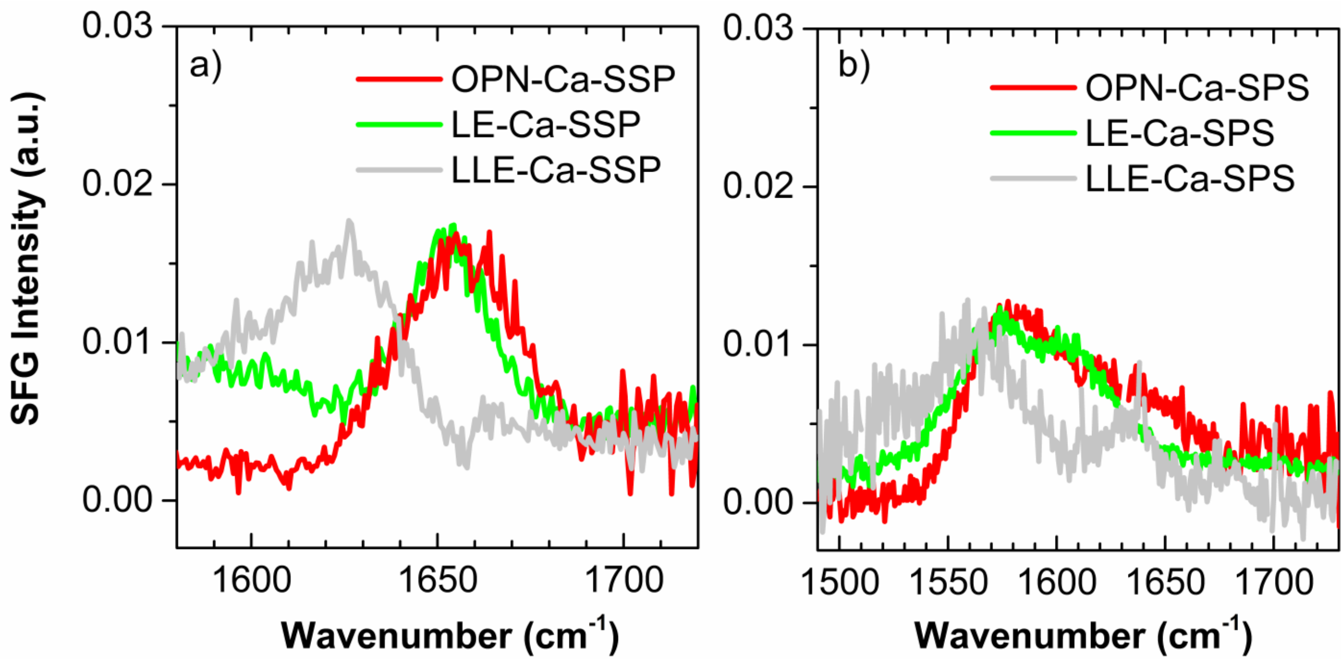

Figure S5. Comparison of different mineralization peptides at the interface with Ca^{2+} ion interaction: red, OPN; green, Leucine-Glutamic peptides with sequence of $E(LE)_9$, labeled as LE; light gray, Leucine-Glutamic peptides with sequence of $E(LLE)_9$, labeled as LLE. The spectra line shape of OPN well mimics that for LE peptides, further implying: like LE sequences[2], OPN peptides adopt a similar β turn motif at the calcium-binding interface.

Table S1. Peak fitting parameters and assignment of amide I SFG spectra (Figure 4b) under SSP polarization combination for OPN peptides at air-water interface, with Ca^{2+} ion interaction, and after CaC_2O_4 mineralization.

	OPN	OPN + Ca^{2+}	OPN+CaC_2O_4
A_1 (a.u.)		2.6	1.4
ω_1 (cm^{-1})<br>ν(C=O) β turn		1652	1657
Γ_1 (cm^{-1})		21	20

Table S2. Peak fitting parameters and assignment of amide I SFG spectra (Figure 4c) under SPS polarization combination for OPN peptides at air-water interface, with Ca^{2+} ion interaction, and after CaC_2O_4 mineralization.

	OPN	OPN + Ca^{2+}	OPN+CaC_2O_4
A_1 (a.u.)		2.7	1.8
ω_1 (cm^{-1})<br>ν_{as} (COO^-)		1572	1571
Γ_1 (cm^{-1})		25	25
A_2 (a.u.)		0.5	1.0
ω_2 (cm^{-1})<br>ν(C=O) β turn		1611	1618
Γ_2 (cm^{-1})		23	20

7. References:

[1] J. F. Moulder, J. Chastain, P. E. Sobol, K. D. Bomben, *Handbook of X-ray Photoelectron Spectroscopy*, Perkin-Elmer, Eden Prairie, MN, USA, **1992**.

[2] H. Lu, A. Schafer, H. Lutz, S. J. Roeters, I. Lieberwirth, R. Munoz-Espi, M. A. Hood, M. Bonn, T. Weidner, *J. Phys. Chem. Lett.* **2019**, *10*, 2170-2174.
